# Phonon Transport Mechanism of Strain-Enhanced Lattice Thermal Conductivity in Penta-NiAs_2_ Monolayer

**DOI:** 10.3390/nano16130828

**Published:** 2026-07-06

**Authors:** Yuqi Zeng, Hongmei Zheng, Linjie Xu, Wenyi Wang, Yi Chen, Ling Pu, Chuanfu Li, Hao Sui, Yangshun Lan, Honggang Zhang

**Affiliations:** Key Laboratory of High Performance Scientific Computation, School of Science, Xihua University, Chengdu 610039, China; zengyuqi@stu.xhu.edu.cn (Y.Z.); zhenghongmei@stu.xhu.edu.cn (H.Z.); xulinjie@stu.xhu.edu.cn (L.X.); wwy301910@stu.xhu.edu.cn (W.W.); chenyi123@stu.xhu.edu.cn (Y.C.); puling@stu.xhu.edu.cn (L.P.); lichuanfu@xhu.edu.cn (C.L.); sh00@xhu.edu.cn (H.S.)

**Keywords:** thermal transport, phonon scattering, Penta-2D materials, DFT

## Abstract

Pentagonal NiAs_2_ is a low-symmetry two-dimensional material relevant to nanoelectronic and thermoelectric applications, but its low lattice thermal conductivity (*κ*) may limit heat dissipation in device-related scenarios. In this work, the strain-dependent lattice thermal transport of monolayer penta-NiAs_2_ is investigated using first-principles calculations combined with the phonon Boltzmann transport equation. The lattice thermal conductivity increases monotonically with tensile strain. Mode-resolved analysis shows that this enhancement mainly originates from the selective reinforcement of the out-of-plane acoustic ZA branch, rather than from a uniform increase in all phonon branches. Tensile strain weakens low-frequency anharmonicity, suppresses phonon scattering, and prolongs the ZA phonon lifetime. Meanwhile, the modified ZA dispersion increases its group velocity, further enhancing its contribution to heat transport. The reduced group velocities of the TA, LA, and most optical branches further limit their contributions to thermal conductivity. The results reveal a ZA-phonon-mediated mechanism for strain-enhanced thermal transport in penta-NiAs_2_ and provide guidance for tuning phonon transport in pentagonal two-dimensional materials.

## 1. Introduction

Pentagonal (Penta-) two-dimensional materials have attracted increasing attention owing to their low-symmetry lattice frameworks and unconventional bonding configurations [1,2,3,4]. Compared with conventional hexagonal two-dimensional materials, pentagonal lattices introduce distinct structural motifs and additional degrees of freedom for property modulation [1,2]. For example, penta-graphene has been predicted to exhibit an intrinsic bandgap and high mechanical strength [5], while penta-PdSe_2_, another representative pentagonal system, exhibits anisotropic electronic transport and excellent environmental stability [6,7]. Recent studies on pentagonal PtS_2_ and ZnS_2_ monolayers have further shown that the pentagonal framework can strongly affect phonon transport and thermoelectric performance [8,9,10]. These observations indicate that pentagonal lattices provide a useful platform for examining how low lattice symmetry and bonding topology regulate phonon-mediated heat transport.

Among various pentagonal two-dimensional materials, penta-NiAs_2_ has been theoretically predicted as a representative low-symmetry system with excellent structural stability and unique electronic properties [11]. Previous studies have shown that penta-NiAs_2_ possesses a narrow direct band gap, high carrier mobility, and pronounced anisotropic transport behavior, making it promising for nanoelectronic and thermoelectric applications [11,12]. However, similar to many pentagonal materials, penta-NiAs_2_ generally exhibits relatively low lattice thermal conductivity (*κ*) due to its strong intrinsic phonon anharmonicity [12]. Although this feature is beneficial for thermoelectric performance, it may limit its use in efficient thermal management and heat dissipation. Therefore, achieving effective regulation and even enhancement of thermal conductivity in penta-NiAs_2_ remains an important yet largely unexplored challenge.

To regulate phonon transport in two-dimensional materials, several phonon-engineering strategies have been explored, such as isotope substitution, defect engineering, nanostructuring, interface engineering, and strain engineering [13,14,15,16]. Isotope disorder and vacancy defects, for instance, can introduce additional phonon-scattering channels and thereby reduce the lattice thermal conductivity of graphene and transition metal dichalcogenides [17,18,19,20]. Nanostructures and heterointerfaces can also suppress heat transport by strengthening boundary and interfacial phonon scattering [14,21]. Nevertheless, these approaches commonly involve impurities, defects, or complex interfaces, which may make the intrinsic relationship between lattice structure and phonon transport less transparent and may also affect the mechanical or electronic performance of the material. Strain engineering, by comparison, offers a more direct and controllable way to tune phonon transport because it can continuously modify lattice geometry without necessarily introducing additional disorder [22,23]. Strain-induced variations in bond lengths, bond angles, and interatomic force constants further reshape phonon dispersion and modify group velocities, thereby regulating phonon anharmonicity and scattering rates [22,24]. Such strain-dependent thermal transport has been reported in graphene, MoS_2_, MoTe_2_, and phosphorene, where lattice thermal conductivity can be strongly modulated and anisotropic thermal responses may emerge [22,23,24,25]. On this basis, applying strain engineering to penta-NiAs_2_ provides a useful route to examine whether its intrinsically low lattice thermal conductivity can be overcome and to clarify the microscopic phonon mechanisms governing thermal transport modulation in pentagonal two-dimensional materials.

In this work, we investigate the strain-dependent lattice thermal transport properties of penta-NiAs_2_ using first-principles calculations combined with phonon Boltzmann transport theory. The results show an increasing trend of thermal conductivity of penta-NiAs_2_ under tensile strain. With increasing tensile strain, the out-of-plane acoustic (ZA) branch gradually becomes the dominant heat-transport channel, in contrast to the weakened contributions from the TA, LA, and optical branches. By analyzing mode-resolved group velocities, phonon lifetimes, Grüneisen parameters, and three-phonon scattering phase space, this work establishes a microscopic connection between tensile-strain-induced flexural phonon modulation and enhanced lattice thermal conductivity in penta-NiAs_2_ monolayer.

## 2. First-Principles Computational Details

First-principles calculations were carried out using the Vienna Ab initio Simulation Pack-age (VASP) with the projector augmented-wave (PAW) method [26,27]. The exchange–correlation interaction was treated within the generalized gradient approximation using the Perdew–Burke–Ernzerhof (PBE) functional [28]. For structural optimization, we used a plane-wave cutoff energy of 800 eV and a 9 × 9 × 1 Monkhorst–Pack k-point mesh [29]. The convergence criteria for the total energy and Hellmann–Feynman forces were set to 10^−7^ eV and 0.01 eV Å^−1^, respectively. A vacuum layer of 20 Å was introduced along the out-of-plane direction to suppress artificial interactions between adjacent periodic images. This strictly parameter-free first-principles scheme avoids the defects of empirical potential methods and can reliably characterize atomic structural deformation under large tensile strain. The transition metal Ni features localized d electrons. Accordingly, we employed the DFT + U scheme with parameters *U* = 3.0 eV and *J* = 0.9 eV to determine the magnetic ground state of the system. The calculated magnetic moment of the system remains zero in all cases, which indicates that the DFT + U correction is not required for this material [30,31]. Phonon dispersions were calculated using the finite-displacement method with a 3 × 3 × 1 supercell, and the force constants were analyzed using the PHONOPY package [32,33]. We applied biaxial tensile strain by uniformly scaling the in-plane lattice constants a and b from 0% to 8%. For each strained configuration, the internal atomic coordinates were fully relaxed while the strained lattice constants were kept fixed. Because the optimized monolayer has equivalent in-plane lattice constants, the biaxial tensile strain was defined as ε = (a − a_0_)/a_0_ × 100%, where a_0_ and a denote the equilibrium and strained in-plane lattice constants, respectively. The optimized structural parameters of the strained systems are summarized in Table 1.

The lattice thermal conductivity (*κ*) is calculated by solving the linearized phonon Boltzmann transport equation using ShengBTE [34]. The second-order force constants, which determine phonon frequencies and group velocities, were obtained using a 5 × 5 × 1 supercell. To describe anharmonic phonon–phonon interactions, we adopt the finite-displacement method to calculate the third-order force constants with a 4 × 4 × 1 supercell and a cutoff up to the third-nearest neighbors [35]. For Brillouin-zone sampling and energy-conservation broadening, we used a 60 × 60 × 1 *q*-point grid and a Gaussian broadening scale parameter of 0.35, respectively. Convergence tests with respect to the *q*-point grid and supercell size confirmed the reliability of the calculated thermal conductivity. To clarify the microscopic origin of the strain-dependent thermal transport, we further extracted the mode-resolved phonon heat capacity, group velocity, and lifetime. According to Equation (3), thermal conductivity relies on the unit cell volume determined by thickness t. We adopted an effective thickness of 2.70 Å, equal to the interlayer distance of NiAs_2_ with van der Waals correction [36]. The same thickness was adopted for all strained structures to ensure a consistent comparison of the strain dependence of *κ*.

## 3. Results

**Figure 1:**
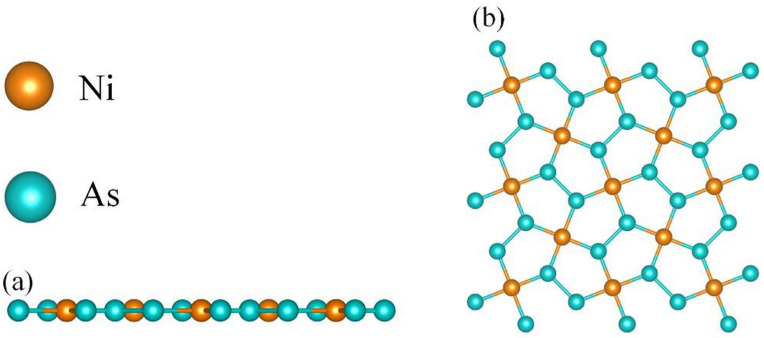
The side view (**a**) and top view (**b**) of the atomic structure diagram of penta-NiAs_2_ under strain-free conditions.

Figure 1 presents the optimized atomic structure of penta-NiAs_2_, which crystallizes in a two-dimensional pentagonal lattice with Pbca symmetry. The side view in Figure 1a shows that the monolayer is nearly planar, whereas the top view in Figure 1b highlights the pentagonal network formed by Ni and As atoms. Owing to the equivalent lattice periodicity along the a and b directions, the optimized lattice constants in the strain-free state are a = b = 5.88 Å. The optimized Ni–As and As–As bond lengths are 2.27 and 2.34 Å, respectively, in good agreement with previous theoretical results [11,12].

**Figure 2:**
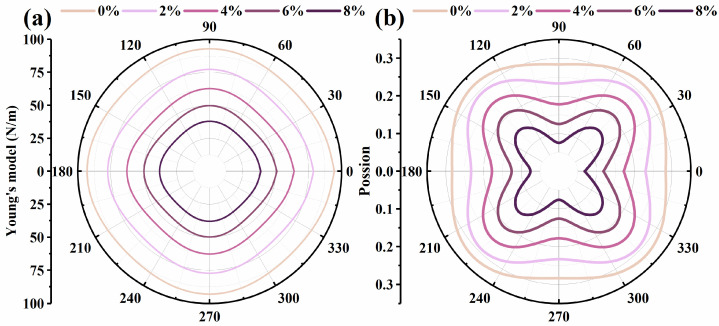
Young’s modulus (**a**) and Poisson’s ratio (**b**) of penta-NiAs_2_ at various angles under different deformations.

For a two-dimensional crystal, the mechanical stability can be evaluated using the Born Huang criteria [37], namely *C*_11_*C*_22_ − $C_{12}^{2}$ > 0 and *C*_66_ > 0. The calculated elastic constants of penta-NiAs_2_ under all considered tensile strains satisfy these criteria, confirming its mechanical stability within the strain range studied. Based on the obtained elastic constants, the angular-dependent Young’s modulus *Y* (*θ*) and Poisson’s ratio *P* (*θ*) were calculated using the following equation [38]:

(1) $$Y(\theta )=\frac{C_{11}C_{22}-C_{12}^{2}}{C_{11}\sin^{4}\theta +C_{22}\cos^{4}\theta +\left(\frac{C_{11}C_{22}-C_{12}^{2}}{C_{66}}-2C_{12}\right)\cos^{2}\theta \sin^{2}\theta },$$

(2) $$P(\theta )=\frac{\left(C_{11}+C_{22}-\frac{C_{11}C_{22}-C_{12}^{2}}{C_{66}}\right)\cos^{2}\theta \sin^{2}\theta -C_{12}\left(\cos^{4}\theta +\sin^{4}\theta \right)}{C_{11}\sin^{4}\theta +C_{22}\cos^{4}\theta +\left(\frac{C_{11}C_{22}-C_{12}^{2}}{C_{66}}-2C_{12}\right)\cos^{2}\theta \sin^{2}\theta },$$

where *θ* is the angle between the loading direction and the lattice vector. For penta-NiAs_2_, the equivalent in-plane lattice periodicity leads to *C*_11_ = *C*_22_, while *C*_12_ and *C*_66_ describe the in-plane coupling and shear responses, respectively. The calculated angular-dependent Young’s modulus and Poisson’s ratio are plotted in Figure 2. As shown in Figure 2a, Young’s modulus exhibits a nearly circular polar profile under all tensile strains, reflecting weak in-plane elastic anisotropy in penta-NiAs_2_. This nearly isotropic response is consistent with the equivalent lattice periodicity along the two in-plane directions. Upon tensile strain, the polar profile contracts continuously, and Young’s modulus decreases from 94.46 N/m in the unstrained structure to 38.71 N/m at 8% strain. The pronounced reduction in Young’s modulus indicates in-plane lattice softening, which can be associated with weakened interatomic force constants under lattice expansion. Since elastic constants describe the long-wavelength mechanical response of the lattice, this softening provides an initial indication that the acoustic phonon dispersion may be substantially modified by tensile strain. The Poisson’s ratio displays a more evident angular dependence, as shown in Figure 2b. Its four-lobed polar profile indicates that the transverse deformation response is more direction-dependent than Young’s modulus. With increasing tensile strain, the average Poisson’s ratio decreases from 0.28 to 0.07, reflecting a weaker transverse response to axial stretching. This reduction points to weakened coupling between longitudinal and transverse lattice deformations. These strain-dependent elastic features therefore provide a mechanical basis for the following discussion of phonon dispersion and thermal transport in strained penta-NiAs_2_.

**Figure 3:**
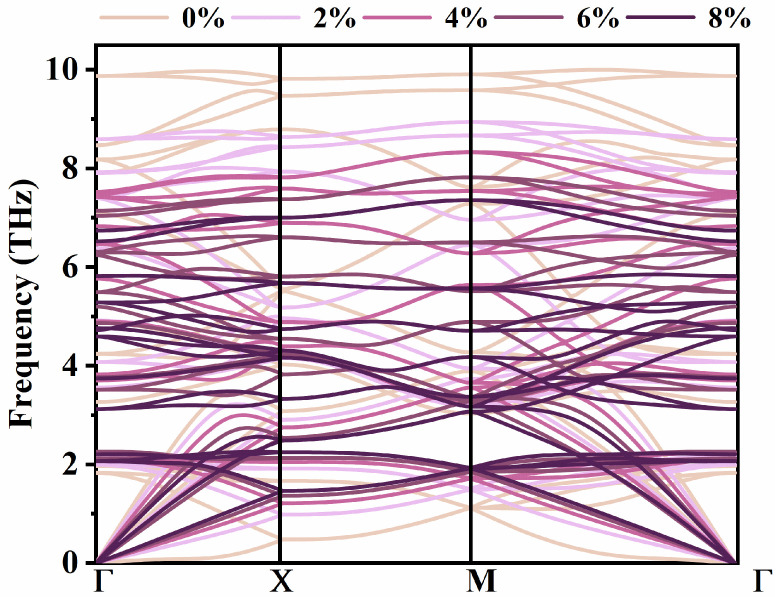
The phonon dispersions of penta-NiAs_2_ under different tensile strains.

To further examine the dynamical stability of penta-NiAs_2_ under tensile strain, phonon dispersions were calculated along the high-symmetry paths in the first Brillouin zone for tensile strains from 0% to 8%, as shown in Figure 3. No imaginary phonon modes are observed at any strain level, confirming that the strained monolayers remain dynamically stable within the considered strain range. Tensile strain leads to a clear upward shift of the ZA branch along the high-symmetry path which indicates a modified flexural phonon dispersion under in-plane lattice expansion. Because phonon group velocity is determined by the slope of the dispersion relation, the strain-induced change in the ZA branch is expected to enhance the group velocity of ZA phonons and thereby affect their contribution to heat transport. In contrast, the TA and LA branches gradually soften under tensile strain, following the reduction in Young’s modules and the weakening of in-plane force constants. Their reduced slopes suggest lower in-plane acoustic phonon velocities. The optical branches also shift downward overall with increasing tensile strain, although the extent of softening varies among different branches. This frequency reduction is consistent with weakened local bond-stretching and bond-bending vibrations involving Ni and As atoms. Such strain-induced changes in both acoustic and optical phonons may further modify acoustic–optical scattering channels, which is important for understanding the subsequent variation in phonon lifetimes and lattice thermal conductivity.

**Figure 4:**
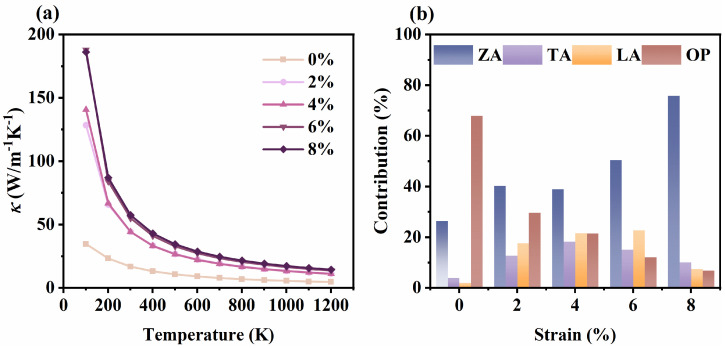
Temperature-dependent lattice thermal conductivity under different tensile strains from 0% to 8% (**a**). Relative contributions of different phonon branches to the total lattice thermal conductivity at 300 K under different tensile strains (**b**).

As shown in Figure 4a, the lattice thermal conductivity (*κ*) of penta-NiAs_2_ decreases with increasing temperature under all strain conditions. This trend is mainly related to stronger anharmonic phonon–phonon scattering at higher temperatures [13,34]. In contrast, tensile strain increases the lattice thermal conductivity over the whole temperature range. At 300 K, *κ* increases from 16.80 W m^−1^ K^−1^ in the unstrained monolayer to 57.40 W m^−1^ K^−1^ at 8% strain. At 1200 K, *κ* also increases from 4.61 to 14.36 W m^−1^ K^−1^ over the same strain range. This enhancement is observed at both low and high temperatures, suggesting that tensile strain changes the main phonon heat-transport channels in penta-NiAs_2_. The branch-resolved lattice thermal conductivity in Figure 4b further shows the evolution of different heat-transport channels with strain. In the unstrained monolayer, the optical branches make the largest contribution to *κ*, while the ZA branch also contributes noticeably. With increasing tensile strain, the ZA contribution increases continuously and becomes dominant at 8% strain. In contrast, the contributions from the TA, LA, and optical branches gradually decrease. This redistribution indicates that the increase in the total lattice thermal conductivity does not arise from a uniform enhancement of all phonon branches, but mainly from the enhanced ZA transport channel. Detailed numerical values and percentage contributions of each phonon branch to thermal conductivity are provided in the Supplementary Materials for reference.

To clarify the microscopic origin of the strain-enhanced lattice thermal conductivity inpenta-NiAs_2_, the mode-resolved transport quantities were analyzed within the phonon Boltzmann transport framework:

(3) $$\kappa =\sum_{qs}C_{V}(qs)v_{g}^{2}(qs)\tau (qs),$$

where *C*_v_(*qs*), *v*_g_(*qs*), and *τ*(*qs*) are the volumetric heat capacity, phonon group velocity, and phonon lifetime of phonon mode (*q*, *s*), respectively. Equation (3) shows that the strain dependence of *κ* is governed by the combined variations in heat capacity, group velocity and lifetime. It is well known that the phonon mean free path satisfies MFP = *v_g_ τ* (Figure S4). Accordingly, we further elaborate the strain-dependent evolution of phonon mean free paths in the Supplementary Materials.

The calculated volumetric heat capacity changes only slightly under tensile strain. It decreases from 2.46 × 10^6^ J m^−3^ K^−1^ in the unstrained monolayer to 2.19 × 10^6^ J m^−3^ K^−1^ at 8% strain, corresponding to a reduction of about 10.84%. This decrease mainly originates from the volume normalization used for the two-dimensional monolayer [13,34]. Tensile strain enlarges the in-plane lattice area, whereas the effective thickness is kept unchanged; consequently, the number of vibrational degrees of freedom per unit volume is slightly reduced. Since this change is opposite to the observed increase in *κ*, the heat-capacity term cannot account for the strain-enhanced thermal conductivity. The following discussion therefore focuses on the strain-induced changes in phonon lifetime and group velocity.

**Figure 5:**
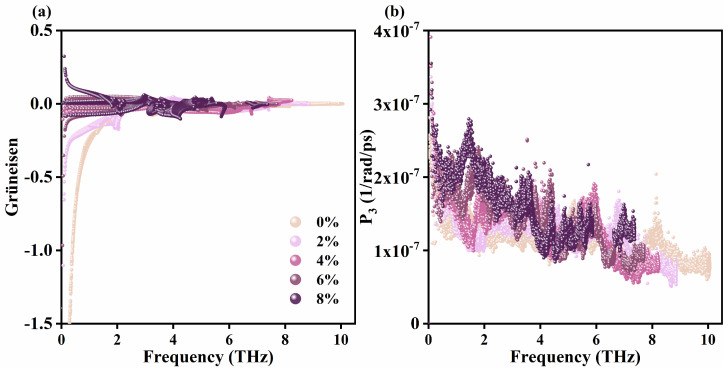
Grüneisen parameters (**a**) and three-phonon scattering phase space *P*_3_ (**b**) of penta-NiAs_2_ under different tensile strains.

The strain dependence of phonon anharmonicity was examined using the Grüneisen parameters and three-phonon scattering phase space, as shown in Figure 5. The Grüneisen parameter is commonly used to describe phonon anharmonicity, and a larger absolute value generally indicates stronger anharmonic interactions. In the unstrained structure, large negative Grüneisen parameters are found in the low-frequency region, especially for the flexural acoustic modes [Figure 5a]. This behavior is often observed for out-of-plane vibrations in two-dimensional materials and suggests strong anharmonicity of the ZA branch in penta-NiAs_2_. Under tensile strain, the magnitude of the negative Grüneisen parameters is clearly reduced, particularly in the low-frequency acoustic region. Since low-frequency acoustic phonons usually play an important role in heat transport, this reduction suggests weakened anharmonicity of the acoustic modes, especially the ZA modes.

The corresponding three-phonon scattering phase space *P*_3_ is shown in Figure 5b. Different from the Grüneisen parameters, *P*_3_ does not decrease monotonically with tensile strain. Instead, tensile strain changes the distribution of available three-phonon scattering channels over the frequency range. In the low-frequency region, *P*_3_ even increases under some tensile-strain conditions, suggesting that additional scattering channels may become available for acoustic phonons. However, *P*_3_ only measures the number of allowed three-phonon processes, whereas the actual scattering rate also depends on the anharmonic interaction strength. Therefore, an increase in *P*_3_ alone does not necessarily lead to stronger phonon scattering. Taken together, the reduced Grüneisen parameters and the nonmonotonic change in *P*_3_ indicate that tensile strain modifies both the strength and the phase space of phonon scattering. Their combined effect will be reflected more directly in the phonon lifetime discussed below.

**Figure 6:**
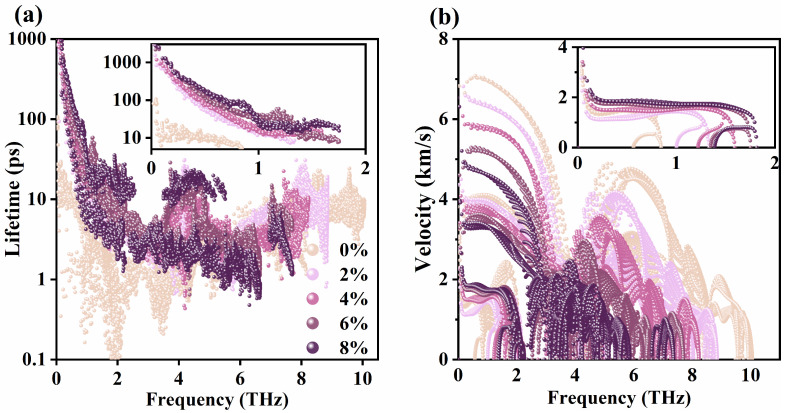
Phonon lifetime *τ* (**a**) and phonon group velocity *v_g_* (**b**) of penta-NiAs_2_ under different tensile strains. Insets show the corresponding data of the out-of-plane ZA phonon branch.

The phonon lifetime was analyzed to further examine the scattering behavior discussed above, as shown in Figure 6a. The main panel presents the lifetimes of all phonon modes, while the inset highlights the ZA branch in the low-frequency region. After tensile strain is applied, the lifetimes of the acoustic phonon branches increase clearly, with the ZA modes showing the most evident change. This trend is consistent with the reduced Grüneisen parameters in the low-frequency region, indicating that tensile strain weakens anharmonic phonon scattering and prolongs the lifetimes of heat-carrying acoustic phonons. The increase in the ZA lifetime is particularly clear in the inset, supporting the view that weakened anharmonicity of flexural vibrations contributes to the enhanced ZA-phonon heat transport (as shown in Figure S2).

Besides the scattering-related lifetime effect, the harmonic contribution was evaluated through the phonon group velocity *v_g_* to examine the harmonic contribution to the strain-dependent thermal conductivity, as shown in Figure 6b. The main panel shows the group velocities of all phonon modes, and the inset gives a clearer view of the ZA branch. With increasing tensile strain from 0% to 8%, the ZA group velocity increases, whereas the group velocities of the TA, LA, and most optical branches generally decrease. This behavior is consistent with the strain-induced changes in phonon dispersion: the ZA branch shifts upward under tensile strain, while the TA, LA, and most optical branches soften. Since *v*_g_ is determined by the slope of the phonon dispersion, the increased ZA group velocity enhances the heat-carrying ability of ZA phonons. When this velocity increase is combined with the prolonged ZA lifetime, the $v_{g}^{2}$*τ* contribution of the ZA modes in Equation (3) is substantially strengthened (as shown in Figure S1). By contrast, the TA, LA, and most optical branches show reduced group velocities under tensile strain, and their contributions to *κ* decrease despite the overall weakening of anharmonic scattering. Therefore, the strain-enhanced lattice thermal conductivity does not originate from a uniform improvement of all phonon modes. Instead, it is mainly driven by the selective reinforcement of the ZA transport channel, which eventually becomes the dominant contributor to the total *κ* under tensile strain.

## 4. Conclusions

In summary, the strain-dependent lattice thermal transport of monolayer penta-NiAs_2_ was investigated using first-principles calculations combined with the phonon Boltzmann transport equation. Penta-NiAs_2_ remains dynamically stable under biaxial tensile strains up to 8%, and its lattice thermal conductivity (*κ*) increases from 16.80 to 57.40 W m^−1^ K^−1^ at 300 K. Tensile strain reduces low-frequency anharmonicity and prolongs the lifetime of ZA phonons, while the modified ZA dispersion increases their group velocity. These two effects jointly enhance the contribution of ZA modes (as shown in Figure 3). In contrast, although the lifetimes of some acoustic modes are also prolonged due to weakened anharmonic scattering, the TA and LA branches show reduced group velocities because of in-plane acoustic phonon softening. Most optical branches also exhibit lower group velocities and contribute less to heat transport. As a result, the increase in total *κ* does not come from a uniform improvement of all phonon modes, but from the selective reinforcement of the ZA branch. This work clarifies the microscopic mechanism of strain-enhanced heat transport in penta-NiAs_2_ and provides a useful reference for strain engineering of phonon transport in pentagonal two-dimensional materials.

## Figures and Tables

**Table 1:** Bond lengths (Å), elastic constants (N/m), Young’s modulus (N/m), and Poisson’s ratio of penta-NiAs_2_ at different lattice constants.

Strain	l_Ni−As_	l_As−As_	*C*_11_	*C*_22_	*C*_66_	*C*_12_	*Y*	*P*
0%	2.27	2.34	102.68	102.68	33.31	29.06	94.46	0.28
2%	2.31	2.36	83.16	83.16	27.53	19.10	78.77	0.23
4%	2.37	2.38	65.96	65.96	22.66	11.33	64.01	0.17
6%	2.42	2.40	51.58	51.58	18.55	6.10	50.86	0.12
8%	2.47	2.41	38.89	38.89	15.05	2.66	38.71	0.07

## Data Availability

Dataset available on request from the authors.

## Supplementary Materials

The following supporting information can be downloaded at: https://www.mdpi.com/article/10.3390/nano16130828/s1, Figure S1: Group velocities of ZA (a), TA (b), LA (c) and OP (d) phonon branches under different tensile; Figure S2. Lifetimes of ZA (a), TA (b), LA (c) and OP (d) phonon branches under different tensile; Figure S3. Contribution and corresponding percentage of each phonon branch to total lattice thermal conductivity at 300 K (a,e), 600 K (b,f), 900 K (c,g) and 1200 K (d,h); Figure S4. Variation of lattice thermal conductivity with phonon mean free path under different strains at 300 K (a), 600 K (b), 900 K (c) and 1200 K (d).
