# Supplementary material for: Phonon Transport Mechanism of Strain-Enhanced Lattice Thermal Conductivity in Penta-NiAs2 Monolayer
Source: Nanomaterials (Basel). 2026 Jul 6;16(13):828. doi: 10.3390/nano16130828 (PMC13362981; doi:10.3390/nano16130828)
Supplement: Supplementary file 1 [file nanomaterials-16-00828-s001.zip › nanomaterials-4369904-supplementary.pdf]

Supplementary Materials

Phonon Transport Mechanism of Strain-Enhanced Lattice Thermal Conductivity in Penta-$NiAs_2$ Monolayer

Yuqi Zeng, Hongmei Zheng, Linjie Xu, Wenyi Wang, Yi Chen, Ling Pu, Chuanfu Li, Hao Sui, Yangshun Lan * and Honggang Zhang *

Key Laboratory of High Performance Scientific Computation, School of Science, Xihua University, Chengdu 610039, China; zengyuqi@stu.xhu.edu.cn (Y.Z.); zhenghongmei@stu.xhu.edu.cn (H.Z.); xulinjie@stu.xhu.edu.cn (L.X.); wwy301910@stu.xhu.edu.cn (W.W.); chenyi123@stu.xhu.edu.cn (Y.C.); puling@stu.xhu.edu.cn (L.P.); lichuanfu@xhu.edu.cn (C.L.); sh00@xhu.edu.cn (H.S.)

* Correspondence: yslansc@foxmail.com (Y.L.); hg.zhang@mail.xhu.edu.cn (H.Z.)

1. Distributions of phonon lifetime and group velocity per phonon branch

To uncover the response mechanism of lattice thermal conductivity (κ), we further perform quantitative analysis resolved by individual phonon branches. Figures S1 and S2 illustrate the frequency-dependent phonon group velocity (v_g) and phonon lifetime (τ) for the out-of-plane acoustic (ZA), transverse acoustic (TA), longitudinal acoustic (LA), and optical (OP) phonon branches under various tensile strains, respectively. It can be clearly observed that the group velocities of all phonons in TA, LA and OP branches decline with increasing tensile strain. By contrast, for the ZA branch, the group velocity only slightly drops at the 2% strain condition, while phonons at all other strain levels exhibit elevated group velocities. As for phonon lifetimes, only optical (OP) phonons show a decreasing trend with growing strain; the lifetimes of ZA, TA and LA phonons all increase progressively as tensile strain rises.

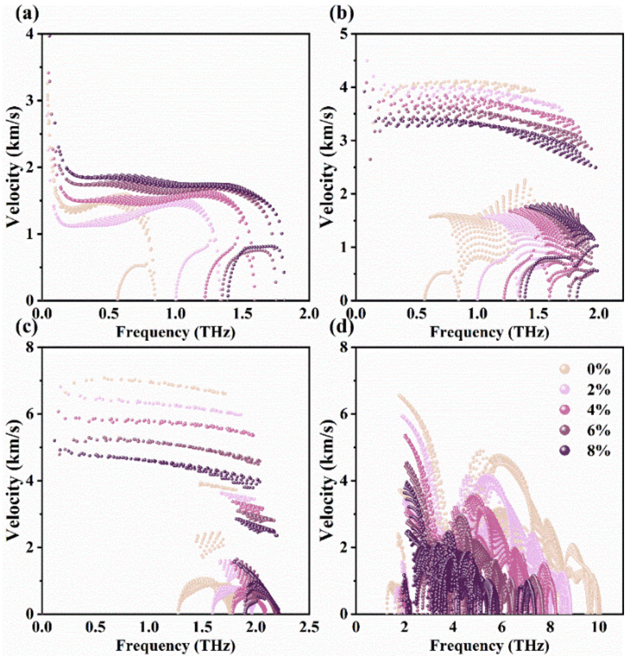

Figure S1. Group velocities of ZA (a), TA (b), LA (c) and OP (d) phonon branches under different tensile.

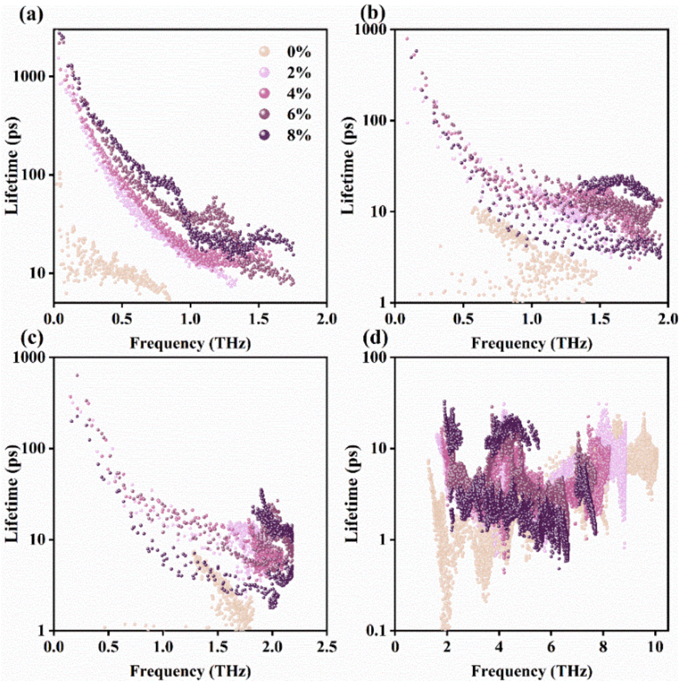

Figure S2. Lifetimes of ZA (a), TA (b), LA (c) and OP (d) phonon branches under different tensile.

2. Thermal conductivity contributions and fraction of each phonon branch

In addition, we quantitatively calculate the absolute contribution and corresponding percentage of ZA, TA, LA and optical phonon branches to the total lattice thermal conductivity at four characteristic temperatures (300 K, 600 K, 900 K, 1200K). The calculated results are presented in Figure S3. The data reveal that acoustic branches (ZA, TA, LA) dominate thermal conduction at all investigated temperatures, among which the ZA branch delivers the largest contribution, whereas optical phonons account for a minor fraction of overall heat transport.

$$\kappa = \sum_{qs} C_V(qs) v_g^2(qs) \tau(qs) \quad \text{(S1)}$$

Equation S1 shows the lattice thermal conductivity is positively correlated with phonon group velocity, phonon lifetime and modal heat capacity (*Cv*). It can be concluded that, despite the continuous reduction of phonon group velocity and modal heat capacity with strain, the extension of phonon lifetime acts as the dominant factor that enhances lattice thermal conductivity under tensile loading.

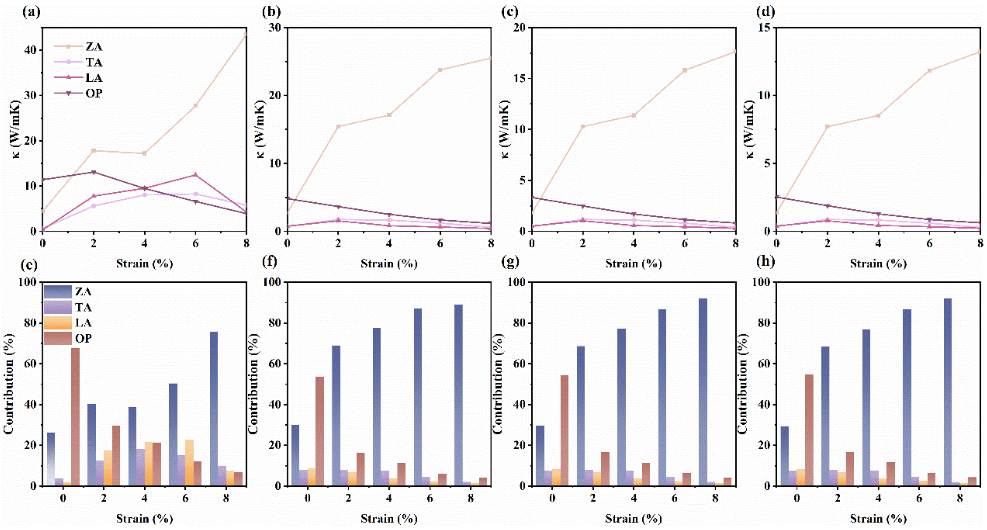

Figure S3. Contribution and corresponding percentage of each phonon branch to total lattice thermal conductivity at 300 K(a、e), 600 K(b、f), 900 K(c、g) and 1200 K(d、h).

3. Phonon mean free paths across temperatures

Figure S4 illustrates the cumulative lattice thermal conductivity as a function of phonon mean free path (MFP) under various tensile strains at 300 K, 600 K, 900 K and 1200 K, and all relevant calculations are implemented following Equation (S2) in this work.

$$\mathrm{MFP} = \sum_{qs} v_g(qs) \tau(qs) \quad \text{(S2)}$$

As widely acknowledged, the thermal conductivity of small-sized samples generally obeys the logarithmic scaling relation κ ~ log L, and our calculated results are consistent with such scaling behavior. Moreover, tensile strain monotonically increases the lattice thermal conductivity over the full range of investigated temperatures. For the strain-free system, heat conduction is dominated by phonons with short mean free paths. Upon applying tensile load, all curves shift toward the region of larger MFP, which indicates that

phonons with long lifetimes and long mean free paths turn into the dominant heat carriers. This observation verifies that the prolonged phonon relaxation time induced by tensile strain acts as the fundamental origin of the enhanced lattice thermal conductivity.

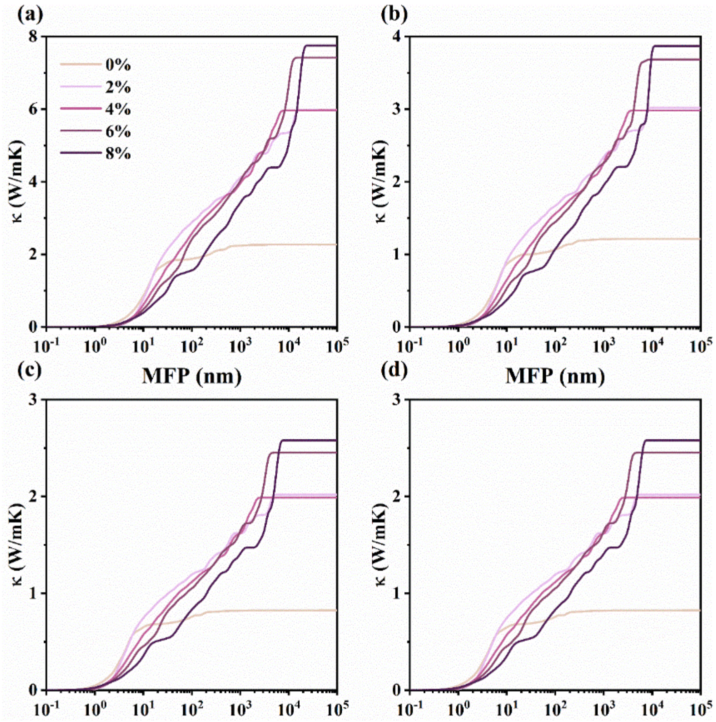

Figure S4. Variation of lattice thermal conductivity with phonon mean free path under different strains at 300 K(a), 600 K(b), 900 K(c) and 1200 K(d).
